# Postoperative chemotherapy significantly improves survival of elderly patients with stage IB‐II non‐small cell lung cancer: A population‐based study

**DOI:** 10.1002/cam4.5834

**Published:** 2023-04-09

**Authors:** Wujianhong Liu, Shangshang Ma, Pingfan Shi, Yanfei Zhang, Ming Li

**Affiliations:** ^1^ Department of Respiratory Medicine, Shanghai Tenth People's Hospital Tongji University School of Medicine Shanghai 200072 China

**Keywords:** elderly, non‐small cell lung cancer, postoperative chemotherapy, stage I‐II, survival analysis

## Abstract

**Background:**

There is scant evidence‐based information about survival benefits of postoperative chemotherapy in elderly patients with early‐stage non‐small cell lung cancer (NSCLC). The purpose of this study is to compare the overall survival (OS) and cancer‐specific survival (CSS) rates of surgery alone versus postoperative chemotherapy in patients aged ≥70 years with stage I‐II NSCLC.

**Methods:**

Elderly patients aged ≥70 years diagnosed with stage I‐II NSCLC were selected from the Surveillance, Epidemiology, and End Results (SEER) database from January 1, 2010 to December 31, 2015. OS and CSS were compared between the two groups utilizing overlap weighting analysis, inverse probability of treatment weight (IPTW), and propensity score matching (PSM).

**Results:**

Of the 7193 included patients with stage I‐II NSCLC who are more than 70 years old, 681 patients (9.5%) received postoperative chemotherapy and 6512 patients (90.5%) received surgery‐alone. Median OS was 77 months in postoperative chemotherapy group versus 79 months in surgery‐alone group (*p* = 0.89). The result of IPTW analysis showed the similar results. The probability of patients choosing chemotherapy increased with the AJCC stage and Grade increasing (*p* < 0.001) and decreased with the growth of age (*p* < 0.001). The results of subgroup analysis showed that the survival rate of stage IA patients decreased significantly after postoperative chemotherapy (*p* < 0.01) while the survival rate of stage IB‐II patients increased significantly (*p* < 0.01). At the same time, we found that patients in the postoperative chemotherapy group tended to have better OS than those in the surgery‐alone group with the grade and tumor size increasing.

**Conclusion:**

The results of this study indicated that postoperative chemotherapy could significantly improve the survival of stage IB‐II NSCLC patients aged ≥70 years, and decrease the survival of stage IA patients.

## Graphical abstract

For patients aged ≥70 years, postoperative chemotherapy can improve the survival rate for those with stage IB and II, and decrease the survival rate for stage IA patients. Therefore, when using chemotherapy, it is necessary to prioritizing the patient's staging along with other vital factors such as age, grade, and other variables in formulating a successful treatment scheme.

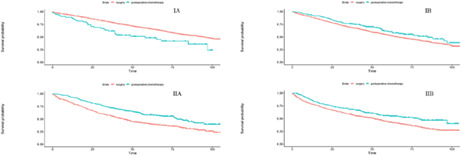

## 1 INTRODUCTION

Lung cancer is one of the leading causes of cancer deaths. According to cancer statistics,^1^ 609,360 patients will be claimed with cancer in the USA by 2022, which suggests about 1700 cancer‐related death will occur every day, including about 350 lung cancer deaths. The main reason is that a considerable number of lung cancer people were already in the advanced stage at the time of diagnosis when they had lost the best chance of treatment.

Non‐small cell lung cancer (NSCLC) occupies a large proportion in most lung cancer cases. In recent years, with the popularization of CT screening and the improvement of health awareness, the proportion of early‐stage NSCLC in all NSCLC cases has increased yearly. From 2010 to 2017, the incidence of stage I NSCLC increased from 10.8 to 13.2 per 100,000 population.^2^ In accordance with the National Comprehensive Cancer Network (NCCN) Guidelines,^3^ postoperative chemotherapy is not recommended for stage IA and not routinely recommended for stage IB NSCLC. In addition, postoperative chemotherapy is recommended for stage II NSCLC.

As the population aging progresses, the proportion of elderly patients has been increasing rapidly. On the other hand, about 50% of newly diagnosed NSCLC cases occurred in people over 70 years.^2^ Many studies have demonstrated that surgery improves the survival and prognosis outcome of elderly patients with stage I or II NSCLC.^4^, ^5^, ^6^, ^7^ But whether postoperative chemotherapy should be recommended in these elderly NSCLC patients remains inconsistent. Although some phase III trials have shown that elderly patients with early NSCLC can obtain a benefit from postoperative chemotherapy,^8^, ^9^, ^10^ no sufficient evidence is available to support their conclusion. Only the 9% of patients in a meta‐analysis of randomized trials were older than 70 years.^11^ Some studies even consider that postoperative chemotherapy is not beneficial, which seems to be related to the increased toxicity such as infection, dehydration, anemia, and age‐related organ dysfunction, and reduced survival rate after limited resection.^12^ Therefore, the impact of postoperative chemotherapy for NSCLC patients aged over 70 years needs to be further defined.

In this study, we analyzed the Surveillance, Epidemiology, and End Results (SEER) database which includes patients with stage I‐II NSCLC patients, and compared the clinical outcomes between postoperative chemotherapy and surgery alone in the aspect of overall survival (OS) and cancer‐specific survival (CSS) in an attempt to provide more strong evidence‐based hints regarding the necessity for postoperative chemotherapy in stage I‐II NSCLC patients.

## 2 PATIENTS AND METHODS

### 2.1 Data source

We collected data from the US National Cancer Institute (NCI) SEER 18 Registries ranging from January 1, 2010 to December 31, 2015. The SEER Database was established in the United States in 1973 and has been an important source of quality information on cancer incidence and survival.^13^ All patients diagnosed with cancer in the previous 5 years had a 90% yearly follow‐up rate.

### 2.2 Patient selection

Patients diagnosed with NSCLC between January 2010 and December 2015 were included in this research. The following were the criteria for inclusion:(1) patients with stage I‐II NSCLC according to the AJCC 7th edition; (2) patients aged ≥70 years; and (3) patients who underwent either surgery‐alone or postoperative chemotherapy. The exclusion criteria were as follows: (1) patients who received radiotherapy; and (2) patients with lacking information regarding age, race, sex, differentiated grade, AJCC stage, tumor location, laterality, and tumor size. Finally, 7193 patients who conformed to the above inclusion and exclusion criteria were enrolled for analysis. Complete information of the patients can be sought from the SEER database.

### 2.3 Covariates

Variables on the patient level and tumor in the process of the analysis were considered. Patient‐level variables included race, sex, age at diagnosis, and the marital status. Tumor‐level variables included the tumor location, tumor size, laterality, AJCC stage and grade. The SEER data dictionary provides a full‐scale description of all the containing variables for your reference.

### 2.4 Objectives

The aim of the study was mainly to compare OS and CSS in elderly patients with stage I‐II NSCLC who had either undergone surgery alone or postoperative chemotherapy. A subgroup analysis was performed to further assess the effectiveness of postoperative chemotherapy.

### 2.5 Statistical methods

We used chi‐square test and Wilcoxon rank sum test to evaluate the correlation between the different treatments and all the contained baseline covariates. The main results included survival rates and survival curves estimated based on the Kaplan–Meier estimator. Logistic regression model was used to assess the choice of postoperative chemotherapy. Cox proportional hazard models were used to determine OS and CSS for both treatment groups, with all baseline covariates adjusted. Three propensity score (PS) models including propensity score matching (PSM), inverse probability of treatment weight (IPTW), and overlapping weighting were used because of the large baseline differences between the two groups. All the covariates in the study between the two groups of patients were matched by using the PS matching method. We use the R package “PSW” to calculate IPTW and overlap weights. The Kaplan–Meier method with log‐rank test was used in all patients for PS models of OS and CSS. In addition, we evaluated the efficacy of postoperative chemotherapy by subgroup analysis of sex, race, AJCC stage and grade, tumor size, and histological type. R version 4.1.1 and IBM SPSS 26 were used for all statistical analyses and values of *p* < 0.05 were considered statistically significant.

## 3 RESULTS

### 3.1 Patient characteristics

**FIGURE 1:**
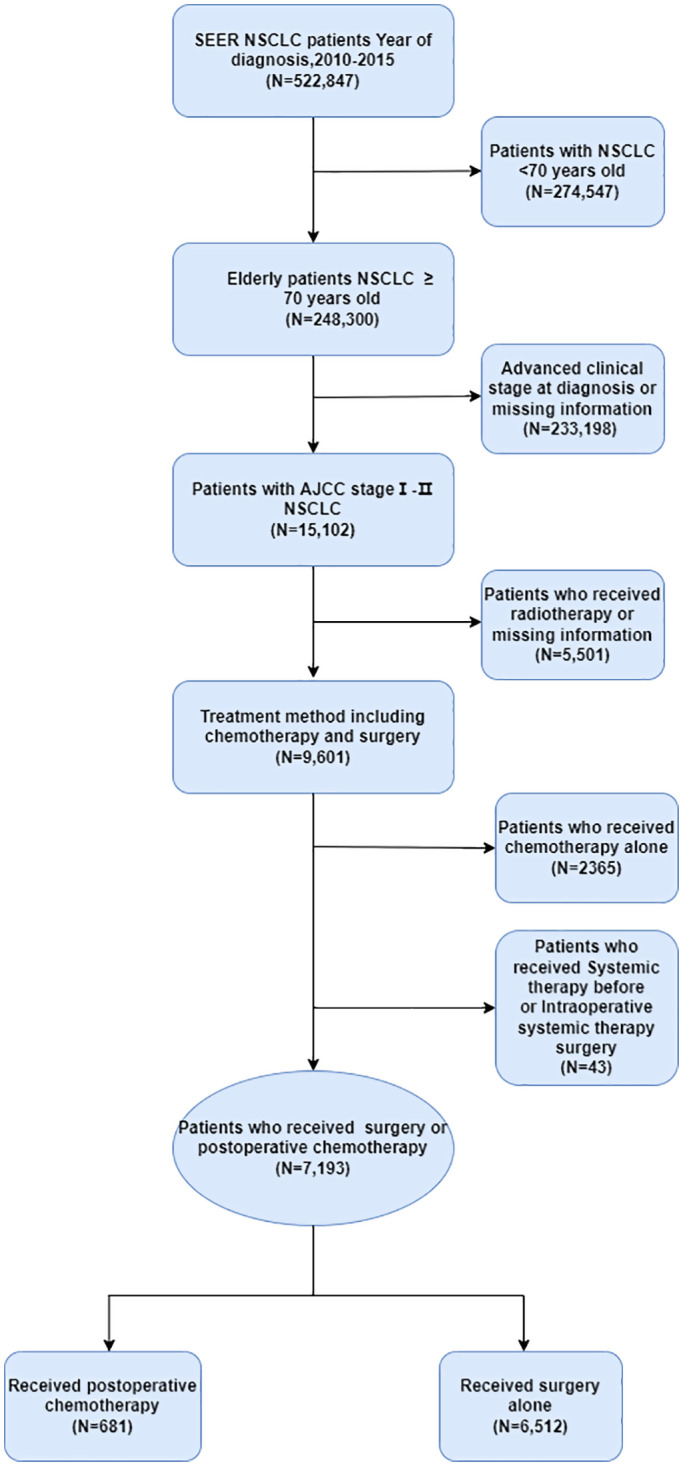
Flowchart of patient selection for the study.

A total of 7193 patients aged ≥70 years with stage I‐II NSCLC were selected from 2010 to 2015. Among these patients, 681 patients (9.5%) received the treatment of postoperative chemotherapy and 6512 patients (90.5%) received surgery alone. Detailed information about the treatment options is shown in Figure 1.

The baseline characteristics of the patients are shown in Table 1. In the unadjusted cohort, there were significant differences in sex, age, AJCC stage and grade, histological type, tumor size, and the marital status between the surgery‐alone and postoperative chemotherapy groups. The unadjusted cohort showed no significant differences in race, primary labeled, and laterality. Both groups achieved a good balance after PS matching. The adjusted cohort shows no significant differences in clinical features.

**TABLE 1:** Demographic and clinical characteristics of the patients with lung cancer.

Characteristics	Before PSM			After PSM			Standardized difference			
	Postoperative chemotherapy (*n* = 681)	Surgery alone (*n* = 6512)	*p*	Postoperative chemotherapy (*n* = 681)	Surgery alone (*n* = 681)	*p*	Unmatched	Matched	IPTW	Overlap weighting
**Sex (%)**			<0.001			0.447	0.148	0.044	0.131	0.062
Male	365 (53.6)	3010 (46.2)		365 (53.6)	350 (51.4)					
Female	316 (46.4)	3502 (53.8)		316 (46.4)	331 (48.6)					
**Race (%)**			0.759			0.431	0.042	0.031	0.031	0.032
White	573 (84.1)	5551 (85.2)		573 (84.1)	562 (82.5)					
Black	51 (7.5)	431 (6.6)		51 (7.5)	45 (6.6)					
Others	55 (8.1)	518 (8.0)		55 (8.1)	72 (10.6)					
Unknown	2 (0.3)	12 (0.2)		2 (0.3)	2 (0.3)					
**Age (%)**			<0.001			0.833	0.47	0.050	0.060	<0.001
70–74	383 (56.2)	2681 (41.2)		383 (56.2)	387 (56.8)					
75–79	236 (34.7)	2186 (33.6)		236 (34.7)	224 (32.9)					
80–84	56 (8.2)	1285 (19.7)		56 (8.2)	64 (9.4)					
≥85	6 (0.9)	360 (5.5)		6 (0.9)	6 (0.9)					
**AJCC(%)**			<0.001			0.98	1.396	0.011	0.128	<0.001
IA	55 (8.1)	3536 (54.3)		55 (8.1)	54 (7.9)					
IB	167 (24.5)	1900 (29.2)		167 (24.5)	170 (25.0)					
II	459 (67.4)	1076 (16.5)		459 (67.4)	457 (67.1)					
**Grade (%)**			<0.001			0.762	0.400	0.074	0.056	<0.001
I	72 (10.6)	1436 (22.1)		72 (10.6)	82 (12.0)					
II	294 (43.2)	2883 (44.3)		294 (43.2)	293 (43.0)					
III	261 (38.3)	1590 (24.4)		261 (38.3)	257 (37.7)					
Undifferentiated	12 (1.8)	63 (1.0)		12 (1.8)	15 (2.2)					
Unknow	42 (6.2)	540 (8.3)		42 (6.2)	34 (5.0)					
**Laterality(%)**			0.479			1	0.051	0.003	0.024	0.026
Right	297 (43.6)	2694 (41.4)		297 (43.6)	298 (43.8)					
Left	384 (56.4)	3816 (58.6)		384 (56.4)	383 (56.2)					
Paired site	0 (0.0)	2 (0.0)		0 (0.0)	0 (0.0)					
**Tumor size (%)**			<0.001			0.731	0.652	0.103	0.142	<0.001
<1 cm	29 (4.3)	658 (10.1)		29 (4.3)	27 (4.0)					
1–2 cm	146 (21.4)	2324 (35.7)		146 (21.4)	159 (23.3)					
2–3 cm	133 (19.5)	1763 (27.1)		133 (19.5)	131 (19.2)					
3–4 cm	115 (16.9)	879 (13.5)		115 (16.9)	105 (15.4)					
4–5 cm	100 (14.7)	411 (6.3)		100 (14.7)	84 (12.3)					
≥5 cm	157 (23.1)	466 (7.2)		157 (23.1)	173 (25.4)					
Unknow	1 (0.1)	11 (0.2)		1 (0.1)	2 (0.3)					
**Primary** **labeled (%)**			0.117			0.707	0.099	0.093	0.012	0.043
Upper lobe	378 (55.5)	3716 (57.1)		378 (55.5)	386 (56.7)					
Middle lobe	45 (6.6)	411 (6.3)		45 (6.6)	33 (4.8)					
Lower	240 (35.2)	2288 (35.1)		240 (35.2)	242 (35.5)					
NOS	4 (0.6)	39 (0.6)		4 (0.6)	7 (1.0)					
Overlapping	11 (1.6)	44 (0.7)		11 (1.6)	11 (1.6)					
Main	3 (0.4)	14 (0.2)		3 (0.4)	2 (0.3)					
**Histologic (%)**			0.023			0.664	0.119	0.068	0.081	<0.001
Large‐cell	17 (2.5)	93 (1.4)		17 (2.5)	13 (1.9)					
Squamous	156 (22.9)	1694 (26.0)		156 (22.9)	166 (24.4)					
Adenocarcinoma	450 (66.1)	4073 (62.5)		450 (66.1)	436 (64.0)					
Others	58 (8.5)	652 (10.0)		58 (8.5)	66 (9.7)					
**Marital (%)**			0.017			0.073	0.165	0.185	0.049	<0.001
Single	47 (6.9)	505 (7.8)		47 (6.9)	57 (8.4)					
Married	410 (60.2)	3623 (55.6)		410 (60.2)	417 (61.2)					
Divorced	77 (11.3)	629 (9.7)		77 (11.3)	57 (8.4)					
Separated	5 (0.7)	37 (0.6)		5 (0.7)	2 (0.3)					
Unmarried or Domestic Partner	1 (0.1)	10 (0.2)		1 (0.1)	0 (0.0)					
Widowed	111 (16.3)	1453 (22.3)		111 (16.3)	131 (19.2)					
Unknow	30 (4.4)	255 (3.9)		30 (4.4)	17 (2.5)					

Abbreviation: PSM, propensity score matching.

### 3.2 Factors affecting treatment selection

Compared with patients aged 70–74 years in surgery‐alone group, the probability of choosing postoperative chemotherapy decreased with age (75–79 years, OR, 0.653; 95% CI, 0.540–0.788; 80–84 years, OR, 0.246; 95% CI, 0.180–0.330; ≥85 years, OR, 0.093; 95% CI, 0.036–0.195, *p* < 0.001). Compared with stage IA, the probability of choosing postoperative chemotherapy increased with the AJCC stage increasing (IB, OR; 5.601; 95% CI, 4.126–7.718; II, OR, 27.471; 95% CI, 20.702–37.154, *p* < 0.001). Compared with Grade I, the probability of choosing postoperative chemotherapy increased with the grade (Grade II, OR, 1.571, 95% CI, 1.186–2.103; Grade III, OR, 1.978; 95% CI, 1.482–2.666, *p* = 0.030).

### 3.3 Survival analysis

**FIGURE 2:**
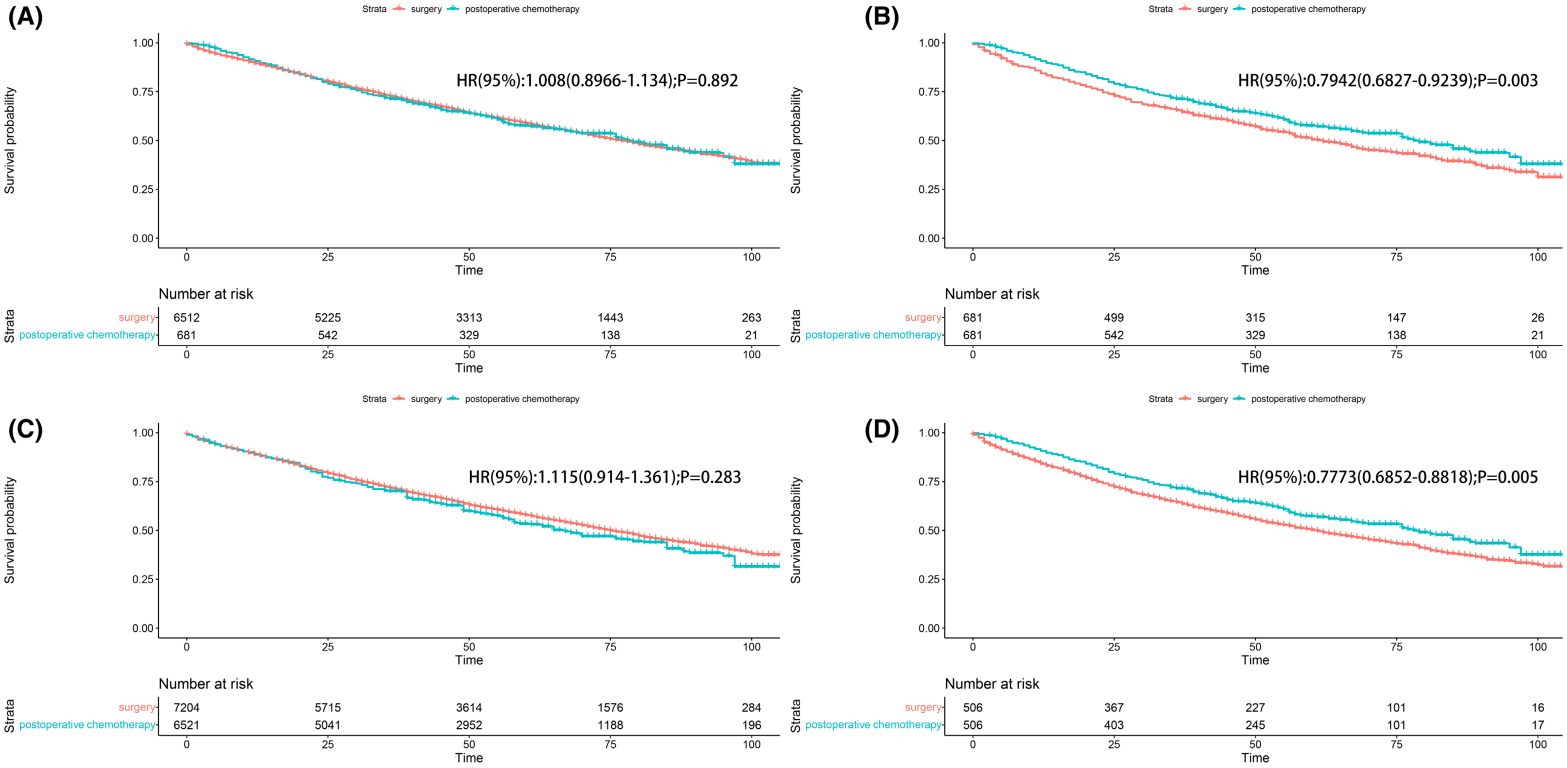
(A) The unmatched analysis; (B) the propensity score matched analysis; (C) the inverse probability of treatment weight‐adjusted analysis; (D) the overlap weighting analysis.

In the unadjusted cohort, the median OS was 77 months for patients in surgery‐alone group versus 79 months for patients in postoperative chemotherapy (*p* = 0.89). The 1‐, 3‐, and 5‐year OS rate was 89.4%, 72.7%, and 58.8% for patients in surgery‐alone group versus 90.7%, 71.2%, and 57.6% for patients in postoperative chemotherapy group, showing no significant difference between the two groups (*p* = 0.89). The IPTW analysis showed the similar results (Table 2). After matching, the median OS in surgery‐alone group was 62 months versus 79 months in postoperative chemotherapy group, showing a better OS outcome in postoperative chemotherapy group (*p* < 0.01). The 1‐, 3‐, and 5‐year OS rate in surgery‐alone group was 84.4%, 65.6%, and 50.4% versus 90.7%, 71.2%, and 57.6% in postoperative chemotherapy group, respectively. The overlap weighting analysis showed the parallel results. The OS survival curves are shown in Figure 2.

**TABLE 2:** Overall survival rate (%) of NSCLC patients between postoperative chemotherapy group and surgery‐alone group.

Year	Unmatched (95% CI)		Matched (95% CI)		IPTW (95% CI)		Overlap weighting (95% CI)	
	Postoperative chemotherapy	Surgery alone	Postoperative chemotherapy	Surgery alone	Postoperative chemotherapy	Surgery alone	Postoperative chemotherapy	Surgery alone
1	90.7 (88.6–92.9)	89.4 (88.7–90.1)	90.7 (88.6–92.9)	84.4 (81.7–87.2)	89.0 (84.7–93.5)	88.8 (88.0–89.6)	90.7 (88.5–93.0)	84.1 (82.5–85.8)
2	79.9 (77.0–83.0)	80.8 (79.9–81.8)	79.9 (77.0–83.0)	73.9 (70.6–77.3)	77.5 (71.9–83.4)	79.9 (78.9–81.0)	80.0 (76.9–83.1)	73.1 (71.1–75.1)
3	71.2 (67.9–74.7)	72.7 (71.6–73.8)	71.2 (67.9–74.7)	65.6 (62.1–69.2)	70.1 (64.1–76.6)	71.7 (70.6–72.9)	71.5 (68.1–75.0)	64.4 (62.3–66.5)
4	64.8 (61.2–68.6)	65.5 (64.3–66.6)	64.8 (61.2–68.6)	58.1 (54.5–62.0)	62.8 (56.4–69.9)	64.5 (63.3–65.7)	64.8 (61.2–68.7)	56.8 (54.7–59.0)
5	57.6 (53.7–61.7)	58.8 (57.5–60.1)	57.6 (53.7–61.7)	50.4 (46.6–54.6)	53.3 (46.1–61.7)	57.8 (56.5–59.1)	57.3 (53.3–61.5)	50.2 (48.0–52.4)

The results of the CSS curve are shown in Figure S1 and Table S1. In the unadjusted cohort, the median CSS in surgery‐alone group was 84 months and 78 months in postoperative chemotherapy group, showing a better outcome in surgery‐alone group (*p* < 0.001). The 1‐, 3‐, and 5‐year incidence rate of CSS was 93.6%, 82.8%, and 74.7% in surgery‐alone groups versus 92.4%, 72.8%, and 68% in postoperative chemotherapy group, respectively. After matching, the median CSS was 75 months in surgery‐alone group versus 78 months in postoperative chemotherapy group, showing no significant difference between the two groups (*p* = 0.079). The 1‐, 3‐, and 5‐year incidence of CSS was 88.7%, 73.5%, and 64.6% in surgery‐alone group versus 92.4%, 72.8%, and 68.0% in postoperative chemotherapy group, respectively, showing similar results in the adjusted cohort as shown by overlapping weight analysis.

**FIGURE 3:**
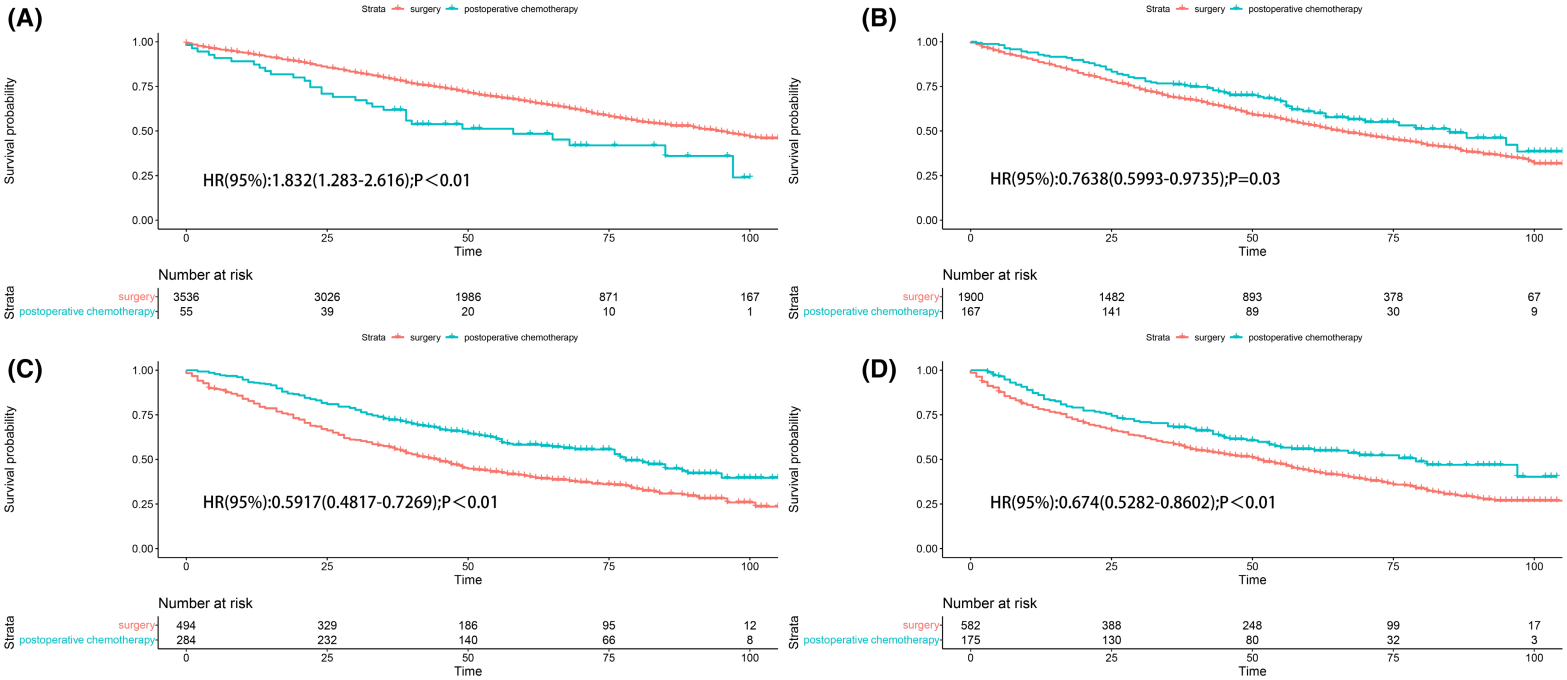
(A) The unmatched analysis of stage IA; (B) the unmatched analysis of stage IB; (C) the unmatched analysis of stage IIA; (D) the unmatched analysis of stage IIB.

**FIGURE 4:**
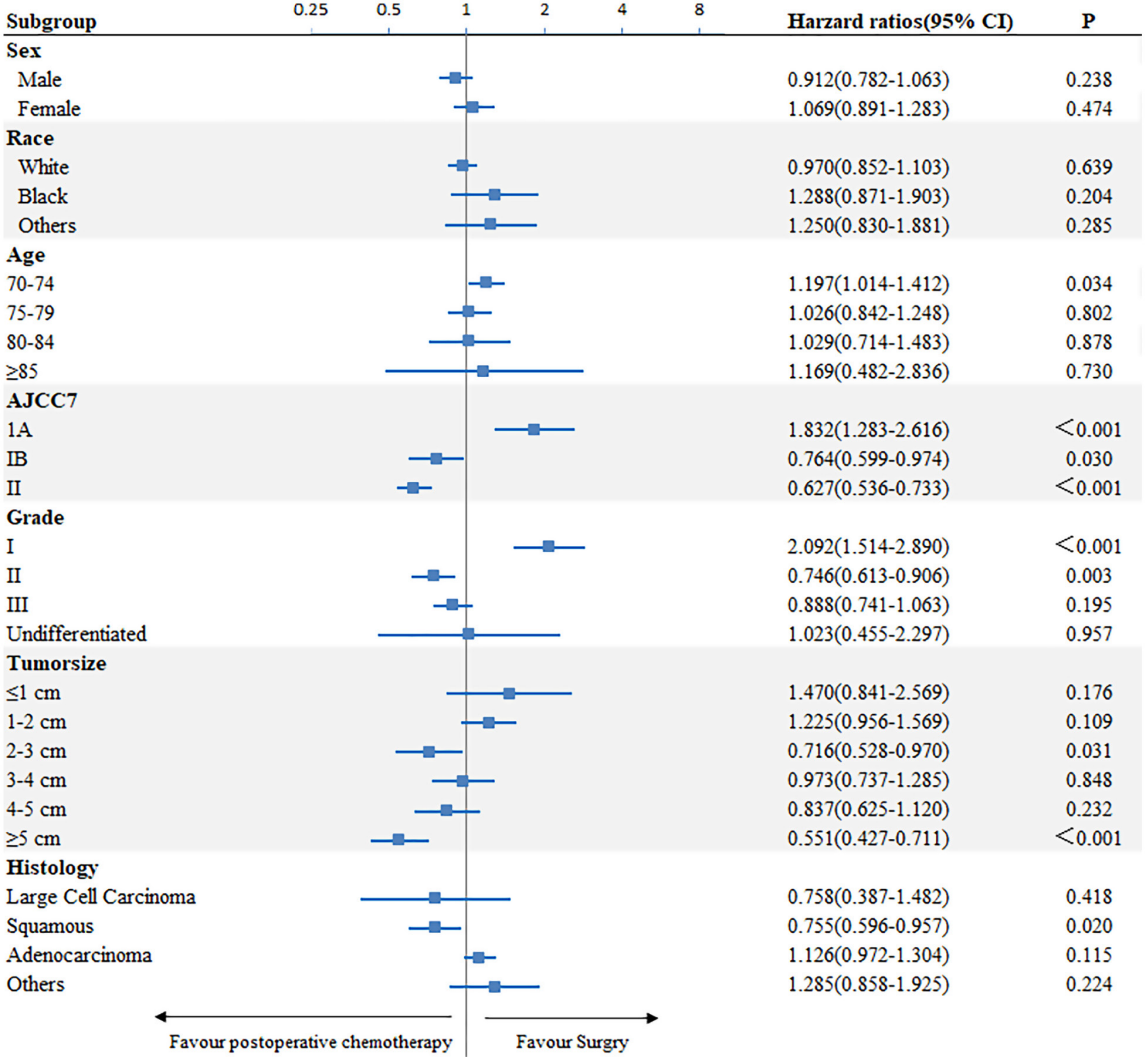
Forest plot depicting unadjusted hazard ratios of postoperative chemotherapy versus surgery for stage I‐II NSCLC.

Knowing that the AJCC stage has a great impact on patient prognosis, we made survival curves of each stage (Figure 3). The results that the survival rate of stage IA patients was decreased after postoperative chemotherapy (HR, 1.832; 95% CI, 1.283–2.616; *p*<0.01) while the survival time of stage IB, IIA, and IIB patients was increased (IB, HR, 0.7638; 95% CI, 0.5993–0.9735, *p* = 0.03; IIA, HR, 0.5917; 95% CI, 0.4817–0.7269, *p* < 0.01; IIB, HR, 0.674; 95% CI, 0.5282–0.8602, *p* < 0.01). The unmatched subgroup analysis also confirmed this conclusion (Figure 4). The grade had the similar features. We found that the survival rate of grade I patients (HR, 1.832; 95% CI, 1.283–2.616, *p* < 0.01) was decreased after postoperative chemotherapy, while the survival rate of grade II and grade III patients was increased. Patients whose histologic is squamous tended to benefit from postoperative chemotherapy (HR, 0.755; 95% CI, 0.596–0.957, *p* < 0.020). In addition, patients who received postoperative chemotherapy tended to have a better OS than those who received surgery alone, showing a significant difference in tumor diameter between 2‐3 cm and ≥5 cm (*p* < 0.05).

## 4 DISCUSSION

The results showed that postoperative chemotherapy significantly improved the survival rate of stage IB and II patients aged ≥70 years, and decreased the survival rate of stage IA patients.

Since the 2004 International Adjuvant Lung Trial (IALT) study,^8^ several phase III trials and meta‐analyses have established the baseline criteria of no chemotherapy for stage IA NSCLC patients, no routine use of chemotherapy for stage IB patients, and use of postoperative chemotherapy for stage II patients.^8^, ^9^, ^10^, ^14^, ^15^, ^16^, ^17^ However, these studies did not include adequate numbers of elderly patients, such as in the JBR. Ten trials in which the age group of over 65 years accounted for only 33%.^9^ Elderly NSCLC patients are considered ineligible for chemotherapy due to their usual progressive organ failure and some complications,^18^ and on the other hand elderly patients often give up chemotherapy after surgery.^19^, ^20^ As a result, there is scant data about adjuvant chemotherapy in patients aged >75 years in the American Society of Clinical Oncology and the Cancer Care Ontario guidelines.^21^ Our findings in patients aged ≥70 years with stage I‐II NSCLC support the guideline recommendation for the whole population for postoperative chemotherapy^3^ and provide additional relevant evidence for clinical treatment choices in this respect.

Previous studies on elderly patients have concluded that postoperative chemotherapy is beneficial. For example, Juan et al.^22^ retrospectively analyzed 3324 patients aged over 65 years with stage II‐IIIA NSCLC who underwent surgery alone or postoperative chemotherapy. The overall survival rate of stage II patients who received postoperative chemotherapy was significantly improved (HR, 0.71; 95%CI, 0.60–0.83). Yamanashi et al.^23^ reviewed 246 NSCLC patients including 102 patients aged >70 years who received postoperative chemotherapy, and found that the relapse‐free survival (RFS) and OS rates in surgery‐alone group were significantly poorer than those in postoperative chemotherapy group (*p* = 0.006; *p* = 0.008). However, the number of samples or patients of these studies is not large enough. In the present study, we included a total of 7193 patients aged ≥70 years with stage I‐II NSCLC, including 681 patients (9.5%) who received postoperative chemotherapy. Our study not only confirmed the findings of the previous studies in elderly patients with IB‐II NSCLC but included a subset of patients with stage IA who were not eligible for postoperative chemotherapy. In addition Hsiao et al.'s retrospective study found that platinum‐based adjuvant chemotherapy (pACT) improved OS in patients with squamous cell carcinoma (75.0 vs. 57.4 months, HR, 0.74, 95% CI, 0.62–0.88, *p* = 0.001), but not adenocarcinoma.^24^ We also found this fact during the subgroup analysis that patients with squamous carcinoma could benefit from postoperative chemotherapy, while patients with adenocarcinoma showed a tendency to be insensitive to postoperative chemotherapy, although the results were not statistically significant. More rigorous prospective randomized studies are required to verify and confirm the results.

Our research had advantages and some disadvantages. It included a large population and reliable data sources. We enrolled 7193 patients aged more than 70 years with stage I‐II NSCLC who underwent surgery alone or postoperative chemotherapy from the SEER database. The present research is a massive cohort study to evaluate the effectiveness of surgery‐alone with that of postoperative chemotherapy in elderly NSCLC patients, with complete clinical data and a low dropout rate. We not only used statistical methods such as IPTW, PSM, and overlap weighting methods to evaluate the effectiveness of postoperative chemotherapy in elderly NSCLC patients but made a subgroup analysis based on the results. Nevertheless, PSM equalizes the distribution of confounders by balancing PS between treatment and control groups, but it reduces the total sample size and discards some important samples because no pairs can be found. IPTW aligns the groups to enroll all populations, making full use of all samples, but introducing a larger bias if the samples themselves carry a larger bias. Overlapping weight compares the populations with the most similar characteristics in the two groups and is less susceptible to extreme weights, but reduces the total sample size even more. Thus, the group with a high proportion of stage IA might show a trend of superiority of surgery alone over postoperative chemotherapy. To further determine which population would benefit from postoperative chemotherapy, we added a stratified analysis based on AJCC staging. Subgroup analysis showed that postoperative chemotherapy significantly increased the survival rate of stage IB and II NSCLC patients, and decreased the survival rate of stage IA NSCLC patients. With the increase of grade and tumor size, patients who received the treatment of postoperative chemotherapy had a better OS tendency than those who underwent surgery alone. The defects of this study lie in the possibility of selective bias because of the retrospective nature of the study and the inherent bias in the use of the SEER database. In addition, the SEER database lacks basic clinical details as for the reasons for patients not choosing chemotherapy, postoperative chemotherapy regimens and associated adverse events, which may affect their prognosis.

In summary, our retrospective analysis suggests that postoperative chemotherapy should not be recommended in stage IA NSCLC but recommended in stage IB and stage II NSCLC patients aged ≥70 years. These findings are consistent with those of several population‐wide studies^9^, ^17^ and provide useful evidence‐based clues for postoperative treatment options in elderly NSCLC patients. Our findings and conclusion still require more rigorous prospective randomized studies to verify and confirm.

## AUTHOR CONTRIBUTIONS

**Wujianhong Liu:** Data curation (equal); formal analysis (equal); methodology (lead); writing – original draft (lead). **Shangshang Ma:** Formal analysis (equal); investigation (equal); project administration (equal); writing – review and editing (lead). **Pingfan Shi:** Data curation (supporting); resources (supporting). **Yanfei Zhang:** Data curation (supporting); methodology (supporting); software (supporting). **Ming Li:** Conceptualization (lead); funding acquisition (lead); resources (supporting); supervision (lead); validation (lead).

## ETHICS STATEMENT

The data from SEER database is de‐identified and publicly available so written informed consent was waived off. The studies including human participants were appraised and supported by Shanghai Tenth People's Hospital.

## Supporting information


Figure S1.

**Table S1.** Cancer‐specific survival rates (%) of postoperative chemotherapy versus surgery in NSCLC patients

**Table S2.** Factors affecting treatment selection

## Data Availability

Publicly available datasets were analyzed in this study. These data can be found in http://seer.gov/data/
