# Supplementary figures and images for: Postoperative chemotherapy significantly improves survival of elderly patients with stage IB‐II non‐small cell lung cancer: A population‐based study
Source: Cancer Med. 2023 Apr 9;12(10):11254–63. doi: 10.1002/cam4.5834 (PMC10242380; doi:10.1002/cam4.5834)

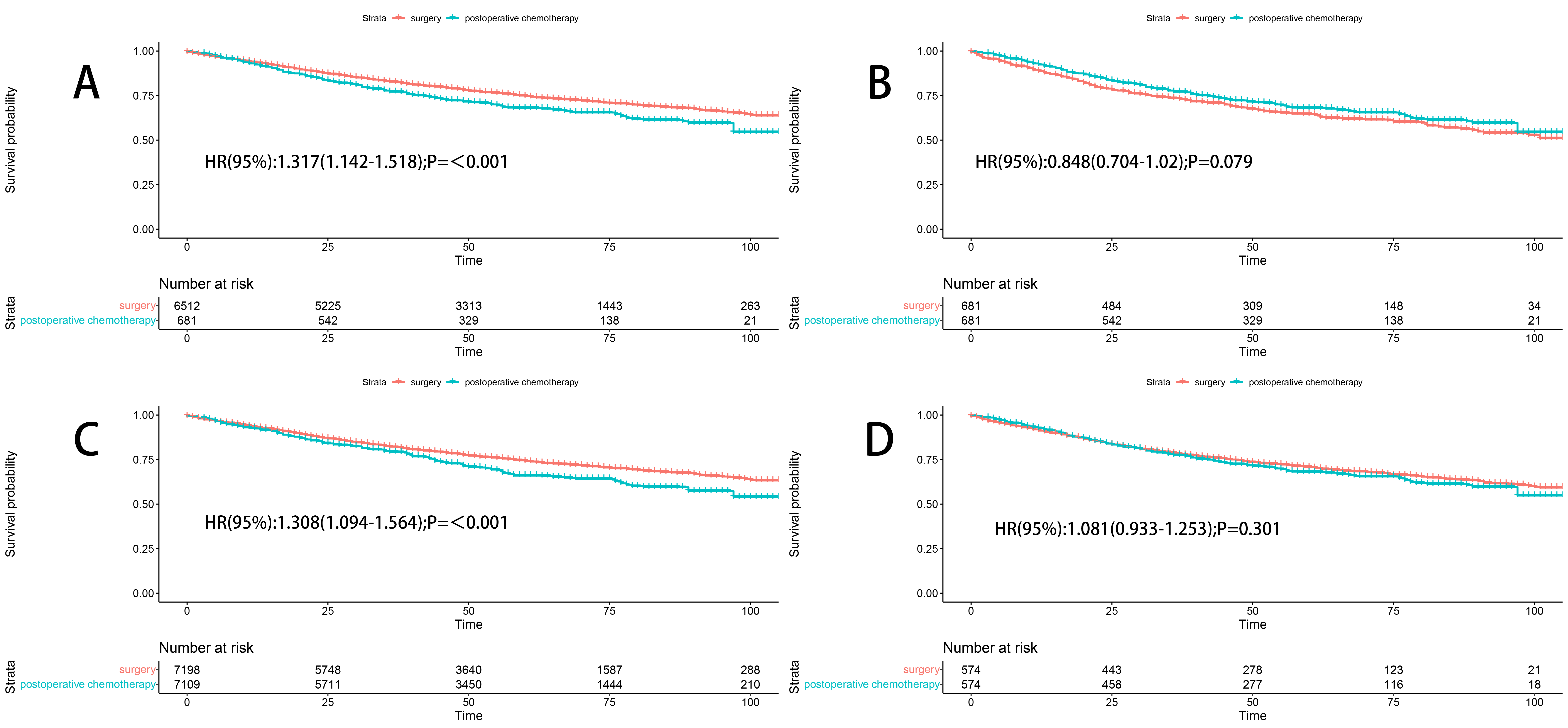

Supplement: Supplementary file 1 — Figure S1. [file CAM4-12-11254-s001.png]
