# Supplementary material for: Postoperative chemotherapy significantly improves survival of elderly patients with stage IB‐II non‐small cell lung cancer: A population‐based study
Source: Cancer Med. 2023 Apr 9;12(10):11254–63. doi: 10.1002/cam4.5834 (PMC10242380; doi:10.1002/cam4.5834)
Supplement: Supplementary file 2 — Table S1. Cancer‐specific survival rates (%) of postoperative chemotherapy versus surgery in NSCLC patients [file CAM4-12-11254-s002.docx]

## Table S1. Cancer-specific Survival Rates (%) of Postoperative chemotherapy versus Surgery in NSCLC Patients

| Year | Unmatched (95% CI) | |  | Matched (95% CI) | |  | IPTW (95% CI) | |  | Overlap Weighting (95% CI) | |
| --- | --- | --- | --- | --- | --- | --- | --- | --- | --- | --- | --- |
|  | postoperative  chemotherapy | Surgery |  | postoperative  chemotherapy | Surgery |  | postoperative  chemotherapy | Surgery |  | postoperative  chemotherapy | Surgery |
| 1 | 92.4(90.5-94.5) | 93.6(93-94.2) |  | 92.4(90.5-94.5) | 88.7(86.3-91.2) |  | 92.5(89.8-95.2) | 93.3(92.7-93.9) |  | 92.6(90.6-94.6) | 91.3(90.2-92.3) |
| 2 | 83.9(81.2-86.8) | 87.7(86.9-88.6) |  | 83.9(81.2-86.8) | 79(75.9-82.2) |  | 84.4(81-87.9) | 87.3(86.4-88.1) |  | 84(81.3-86.9) | 84(82.7-85.4) |
| 3 | 77.2(74-80.5) | 82.8(81.8-83.7) |  | 77.2(74-80.5) | 73.5(70.1-77) |  | 79.3(75.6-83.2) | 82.3(81.3-83.2) |  | 77.4(74.2-80.7) | 78.6(77.2-80.1) |
| 4 | 72.3(68.9-75.9) | 78.6(77.5-79.7) |  | 72.3(68.9-75.9) | 68.3(64.7-72.1) |  | 72.8(68.3-77.6) | 78.1(77-79.2) |  | 72.4(69-76) | 74.2(72.7-75.8) |
| 5 | 68(64.3-71.9) | 74.7(73.5-75.9) |  | 68(64.3-71.9) | 64.6(60.7-68.6) |  | 66(60.7-71.8) | 74.2(73-75.4) |  | 67.9(64.2-71.9) | 70.7(69.1-72.4) |
