# Supplementary material for: Postoperative chemotherapy significantly improves survival of elderly patients with stage IB‐II non‐small cell lung cancer: A population‐based study
Source: Cancer Med. 2023 Apr 9;12(10):11254–63. doi: 10.1002/cam4.5834 (PMC10242380; doi:10.1002/cam4.5834)
Supplement: Supplementary file 3 — Table S2. Factors affecting treatment selection [file CAM4-12-11254-s003.docx]

**TableS2.Factors affecting treatment selection**

| **Characteristics** | **N** | **OR** | **95% CI** | **P** |
| --- | --- | --- | --- | --- |
| **Age** |  |  |  | < 0.001 |
| 70-74 | 3064 | Reference | - |  |
| 75-79 | 2422 | 0.653 | 0.540-0.788 |  |
| 80-84 | 1341 | 0.246 | 0.180-0.330 |  |
| ≥85 | 366 | 0.093 | 0.036-0.195 |  |
| **Sex** |  | - | - | 0.110 |
| **Race** |  | - | - | 0.432 |
| **AJCC** |  |  |  | < 0.001 |
| ⅠA | 3591 | Reference | - |  |
| ⅠB | 2067 | 5.601 | 4.126-7.718 |  |
| Ⅱ | 1535 | 27.471 | 20.702-37.154 |  |
| **Grade** |  |  |  | 0.030 |
| Ⅰ | 1508 | Reference | - |  |
| Ⅱ | 3177 | 1.571 | 1.186-2.103 |  |
| Ⅲ | 1851 | 1.978 | 1.482-2.666 |  |
| **Laterality** |  | - | - | 0.481 |
| **Histologic** |  | - | - | 0.283 |
| **Tumor size** |  | - | - | 0.260 |
| **Marital** |  | - | - | 0.570 |
